# Pluripotent Transcription Factors Possess Distinct Roles in Normal versus Transformed Human Stem Cells

**DOI:** 10.1371/journal.pone.0008065

**Published:** 2009-11-30

**Authors:** Junfeng Ji, Tamra E. Werbowetski-Ogilvie, Bonan Zhong, Seok-Ho Hong, Mickie Bhatia

**Affiliations:** 1 Stem Cell and Cancer Research Institute, McMaster University, Hamilton, Ontario, Canada; 2 Ontario Institute for Cancer Research, Toronto, Ontario, Canada; 3 Clinical Research Division, Fred Hutchinson Cancer Research Center, Seattle, Washington, United States of America; Cincinnati Children's Hospital Medical Center, United States of America

## Abstract

**Background:**

Cancer and normal stem cells (SCs) share proliferative properties of self-renewal and expression of key transcription factors (TFs). Despite similar TF identities, the functional role of specific TFs responsible for retaining SC state has yet to be examined in cancer.

**Methodology/Principal Findings:**

Here, we compare the role of *Oct4* and *Nanog*, two-core pluripotent TFs, in transformed (t-hPSCs), and normal human pluripotent stem cells (hPSCs). Unlike normal SCs, self-renewal and survival of t-hPSCs were found to be independent of Oct4. In contrast, t-hPSCs exhibit hypersensitivity to reduction in Nanog and demonstrate complete loss of self-renewal coupled with apoptosis. Dual and sequential knockdown of Oct4 and Nanog revealed that sensitivity of t-hPSCs to Nanog was Oct4 dependent.

**Conclusions/Significance:**

Our study indicates a bifurcation for the role of two-core SC and cancer related TFs in self-renewal and survival processes. We suggest that the divergent roles of these TFs establish a paradigm to develop novel therapeutics towards selective destruction of aggressive tumors harboring cancer stem cells (CSCs) with similar molecular signatures.

## Introduction

Cancer cells share a variety of properties with normal SCs including self-renewal capacity, but lack the ability to differentiate and undergo apoptosis in a similar fashion to normal SCs. Cell populations have been identified in a variety of human cancers that possess self-renewal capacity, but are also capable of initiating tumor heterogeneity in xenograft models [1], [2], [3], [4], [5], [6]. These properties, along with phenotypic resemblance to normal SCs, define the term Cancer Stem Cell (CSC) [7], [8] and perpetuate the notion that CSCs may capitalize on molecular machinery controlling normal SC function for maintaining oncogenic properties. For example, *Bmi-1*, a polycomb group (PcG) gene, was shown to be essential for both normal and leukemic mouse SC proliferation [9]. Aside from this work, little is known about the functional relevance of genetic determinants to CSCs versus their normal SC equivalents.

Molecular control of self-renewal is well established in the embryonic stem cell (ESC) system and has been found to be governed by a core set of TFs that maintain the undifferentiated ground state [10]. These factors that include Octamer4 (*Oct4)* [11], [12] and *Nanog* [12], [13], [14] have recently been associated with highly aggressive adult tumors [15], [16], [17], [18], [19], [20]. These observations suggest that factors controlling robust self-renewal unique to ESCs may be important for aggressive somatic tumor growth [21]. To this point, overexpression of *Oct4* is sufficient to induce dysplastic growth in adult mouse epithelium [22] and enhance the malignant potential of ESC-derived germ cell tumors [19]. Similarly, Nanog expression has also been detected in a variety of human neoplasms [23], [24], [25], [26], [27], [28], [29]. Downregulation of Nanog has recently been shown to inhibit prostate, breast and colon tumor development both *in vitro* and *in vivo* [15]. However, the functional and mechanistic roles of *Oct4 and Nanog* in CSCs vs. normal SCs are unknown.

hPSCs with features of neoplastic progression including aberrant self-renewal and resistance to differentiation amounting to enhanced tumorigenic potential have recently been characterized [30]. To determine the role of core pluripotent TFs in human SC transformation, we directly compared the effect of Oct4 and Nanog downregulation on self-renewal of normal vs. transformed hPSCs. t-hPSCs, unlike their normal counterparts, are independent of Oct4 for self-renewal, pluripotency and survival. Both cell types require Nanog for SC state maintenance, but t-hPSCs exhibit an unprecedented dependency on Nanog for self-renewal and cell survival. Our study establishes a paradigm by which functional divergence of pluripotent TFs from the normal SC state accompanies transformation and could therefore be used to develop therapies targeting somatic CSCs in aggressive tumors.

## Results

### Downregulation of Oct4 Does Not Alter Self-Renewal or Survival of t-hPSCs

To determine the functional relevance of Oct4 in normal and transformed hSCs, we stably knocked down Oct4 levels in both normal hPSCs and t-hPSCs using shRNA. Quantification of Oct4 downregulation by flow cytometry demonstrated effective knockdown in both cell types (Figure S1A–H). This was determined by frequency of Oct4+ cells (Figure S1A–C, E–G) and the number of Oct4 molecules/cell measured by mean fluorescent intensity (Figure S1D and H).

**Figure 1:**
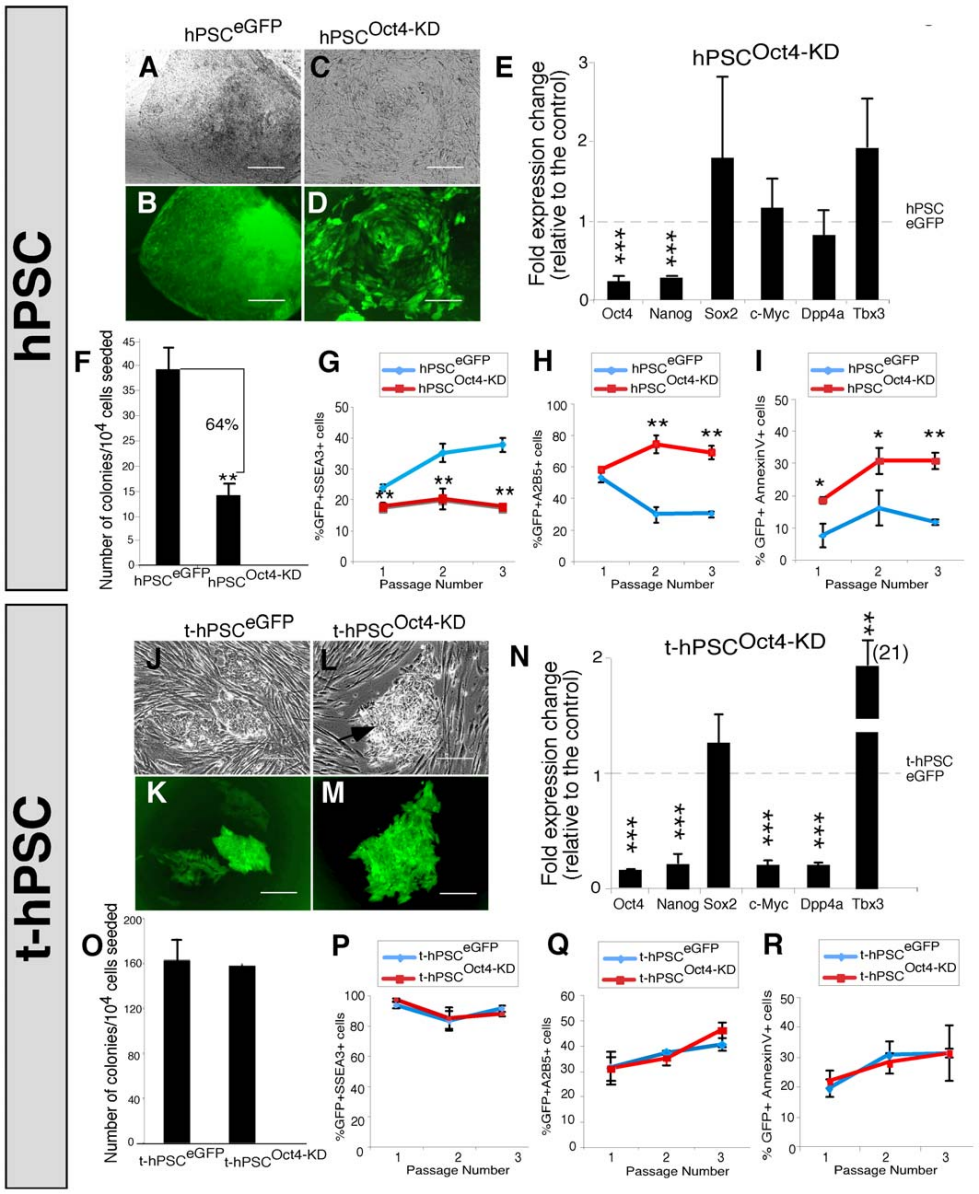
Oct4 knockdown does not affect self-renewal, differentiation and survival of t-hPSCs. (A–D) Representative images of colonies generated from GFP^+^SSEA3^+^ cells 11 days post-sort from control (A and B) and Oct4 knockdown (C and D) normal hPSCs. Scale bar = 100 µm, n = 3. Note the compact appearance and well-defined border of the typical hPSC colony in A and B. In contrast, the cells within the more differentiated colony in C and D exhibit a higher cytoplasm to nuclear ratio, are less compact, and do not form a well-defined colony border. (E) qPCR of fold changes in Oct4, Nanog, Sox2, c-Myc, Dpp4a, and Tbx3 transcripts in Oct4 knockdown hPSCs relative to hPSC ^eGFP^ controls. Bar graphs represent mean values ± SEM, n = 3, ***, p<0.001. (F) Clonogenic self-renewal of SSEA3^+^ cells isolated from control (39.3±4.3×10^4^) and Oct4 knockdown (14±2.3×10^4^) hPSCs. 1×10^4^ GFP^+^SSEA3^+^ cells were isolated from hPSCs 2 days after transduction with eGFP control and Oct4 knockdown lentiviral vectors and seeded on ihdFs. GFP^+^ colonies were scored 9 days after seeding. Bar graphs represent mean values ± SEM, n = 3. **, p<0.01. (G–I) Frequency of GFP^+^SSEA3^+^ (G) GFP+A2B5+ (H) and GFP+AnnexinV+ (I) cells for three passages of culture derived from sorted GFP^+^SSEA3^+^ fractions of control and Oct4 knockdown hPSCs. N = 3 for each. Line graphs represent mean values ± SEM. * p<0.05, ** p<0.01. (J–M) Representative images of colonies generated from sorted GFP^+^ cells 6 days post-sort from control (J–K) and Oct4 knockdown (L–M) t-hPSCs. Scale bar = 100 µm, n = 5. Arrow denotes the typical appearance of tightly packed undifferentiated colonies. (N) qPCR of fold changes in Oct4, Nanog, Sox2, c-Myc, Dpp4a, and Tbx3 transcripts in Oct4 knockdown t-hPSCs relative to t-hPSC ^eGFP^ control cells. Bar graphs represent mean values ± SEM, n = 3, **, p<0.01, ***, p<0.001. (O) Clonogencity of GFP^+^ cells isolated from control and Oct4 knockdown t-hPSCs. 1×10^4^ GFP^+^ cells were sorted from t-hPSCs 4 days after transduction with eGFP control and Oct4 knockdown lentiviral vectors and seeded on ihdFs. GFP^+^ Colonies were scored 6 days after. Bar graphs represent mean values ± SEM, n = 3. (P–R) Frequency of GFP^+^SSEA3^+^ (P) GFP+A2B5+ (Q) and GFP+AnnexinV+ (R) cells for three passages of culture derived from sorted GFP^+^ fractions of control and Oct4 knockdown t-hPSCs. P, n = 9. Q, n = 5, R, n = 3. Line graphs represent mean values ± SEM.

Consistent with previous reports [31], [32] hPSC colonies differentiated 7 days following Oct4 depletion (Figure S1I–L). However, hPSC cultures are morphologically, phenotypically, and functionally heterogeneous, and are re-established by rare colony-initiating cells (CICs) enriched in the SSEA3+ fraction [33]. To dissect the role of Oct4 in this clonogenic subpopulation, we isolated normal hPSCs based on green fluorescent protein (GFP) in combination with the undifferentiated hSC marker SSEA3 and quantitatively compared subsequent colony growth (Figure S2). Oct4 downregulation resulted in visible differentiation of hPSC colonies (Figure 1A–D).

To investigate the molecular mechanisms associated with Oct4 depletion in hPSCs, we compared changes in transcript levels of Oct4, Nanog, SRY (sex determining region Y)-box 2 (Sox2), V-myc myelocytomatosis viral oncogene homolog (avian) (c-Myc), dipeptidyl-peptidase 4a (Dpp4a) and T-box 3 (Tbx3), all implicated in pluripotent stem cell maintenance (Table S2) [12], [34]. As expected, lentiviral shRNA transduction of Oct4 significantly reduced Oct4 but also downregulated Nanog transcripts in SSEA3+ hPSCs (Figure 1E). However, Sox2, c-Myc and Tbx3 levels showed slight, non-significant increases following Oct4 dysregulation (Figure 1E) while Dpp4a levels were minimally decreased (Figure 1E). Taken together, our results confirm the previously established role of Oct4 in differentially regulating gene expression in normal hPSCs, and the central importance of these factors in maintaining the pluripotent state [12], [31], [34], [35], [36].

In addition to the molecular changes seen following Oct4 knockdown, we dissected the biological effects on the self-renewing hPSC SSEA3+ fraction. Oct4 downregulation reduced the total number of clonogenic self-renewing cells (CICs) by 64% compared with cells transduced with the eGFP control vector (Figure 1F). In addition, Oct4 downregulation significantly decreased the frequency of undifferentiated SSEA3^+^ cells and increased the frequency of the neural precursor marker, A2B5, compared with control eGFP cells (Figure 1G–H). Oct4 knockdown also induced cell death as demonstrated by AnnexinV^+^ staining (Figure 1I). These results show that Oct4 regulates a differentiation response in the self-renewing fraction of normal hPSCs and is therefore required for maintenance and survival of the hPSC undifferentiated state.

Unlike normal hPSCs, t-hPSCs are less morphologically and phenotypically heterogeneous demonstrated by ubiquitous expression of SSEA3 throughout the culture, and do not require the fibroblast-like cell supportive niche [30]. To evaluate the functional role of Oct4 in t-hPSCs, we stably knocked down Oct4 by shRNA and then isolated fractions based on green fluorescent protein (GFP) (Figure S2). Surprisingly, both t-hPSC bulk cultures (Figure S1M–P) and GFP+ fractions (Figure 1J–M) appeared completely unaffected by Oct4 dysregulation. Undifferentiated colonies were phenotypically similar to both the control and parental t-hPSCs (Figure 1J–M).

Comparable to normal hPSCs, Oct4 reduction in t-hPSCs resulted in a significant and predictable reduction in Oct4 as well as a similar decline in Nanog and a small increase in Sox2 levels (Figure 1N). Along with a substantial increase in Tbx3 expression, both c-Myc and Dpp4a were significantly reduced in Oct4 t-hPSCs. (Fig. 1N). Differential regulation of c-Myc, Dpp4a and Tbx3 reveals a molecular distinction in response to Oct4 depletion between t-hPSCs and hPSCs that may be, at least in part, responsible for the lack of response of t-hPSCs to Oct4 dysregulation.

Although there were no discernible changes in t-hPSC colonies following Oct4 knockdown, we further assessed the functional relevance of Oct4 to t-hPSC clonogenic self-renewal, differentiation and survival. Unlike normal hPSCs, downregulation of Oct4 had no effect on colony formation (Figure 1O) or the frequencies of SSEA3^+^, A2B5^+^, and AnnexinV^+^ cells over multiple passages (Figure 1P–R). The inability to alter self-renewal in t-hPSCs persisted even four months after Oct4 depletion (Figure S1Q–T). These data demonstrate that Oct4 is dispensable for the self-renewal and survival of t-hPSCs, but is critical to normal hPSC maintenance. Despite the established key role of Oct4 in sustaining pluripotency, our results provide direct evidence for the functional divergence of Oct4 from the pluripotent state following transformation.

### Oct4 Is Not Required for Pluripotency and Tumorigenicity of t-hPSCs

**Figure 2:**
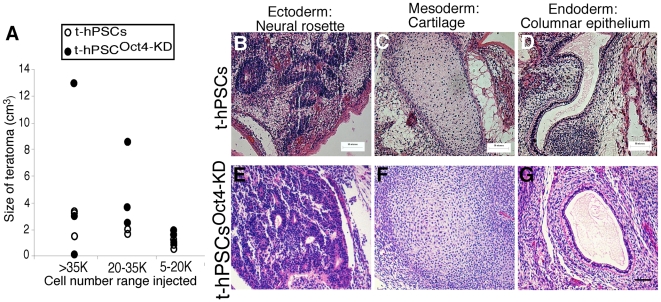
Downregulation of Oct4 has no effect on t-hPSC pluripotency or tumor-initiating cell capacity. (A) Summary of teratoma formation from control and Oct4 knockdown t-hPSCs at serial injected cell doses. Cell doses of 7.5×10^3^, 2.4×10^4^, and 6.6×10^4^ t-hPSCs and 1.5×10^4^, 3.3×10^4^ and 6.6×10^4^ Oct4 knockdown t-hPSCs were injected into the testis capsules of NOD/SCID mice. Mice were sacrificed 6 weeks after injection. Teratoma size was measured and plotted relative to injected cell doses. N = 7. (B–G) Representative histology of teratomas formed in NOD/SCID mice testes 6 weeks following injection of control t-hPSCs (B–D, upper panel) or Oct4 knockdown t-hPSCs (E–G, bottom panel). Tissues representing all three embryonic germ layers including ectoderm (neural rosettes, B and E, left panels), mesoderm (cartilage, C and F, middle panels), and endoderm (columnar epithelium, D and G, right panels) are shown. Scale bar = 50 µm.

hPSC pluripotency is determined *in vivo* by the presence of all 3 germ layers in teratomas formed in human-mouse xenografts. Teratomas are formed from a rare subset of cells present at a frequency of 1∶17500 cells in normal hPSCs [30]. In contrast, we have recently shown that t-hPSCs are highly enriched for teratoma-initiating cells (TICs) with a frequency of 1∶800 and give rise to teratomas containing clusters of Oct4 positive cells [30]. Oct4 expression has been associated with more aggressive tumors and is suggested to be a malignant teratocarcinoma marker *in vivo* [19], [37], [38]. To determine whether Oct4 is related to the higher TIC frequency and capacity of t-hPSCs, we injected t-hPSCs depleted in Oct4 at different cell doses into NOD-SCID mice. All mice (9/9 mice), regardless of limiting dose, developed teratomas (Figure 2A; Table S1). Teratomas generated from both Oct4 depleted and control t-hPSCs consisted of tissues representing all three germ layers (Figure 2B–G; Table S1). Additionally, in 7 of 8 Oct-4 depleted teratomas, Oct4 staining was absent demonstrating sustained Oct4 knockdown in these tumors (data not shown). Furthermore, these teratomas were similar in size to controls suggesting that Oct4 does not affect t-hPSC proliferation or TIC capacity *in vivo* (Figure 2A; Table S1). These results demonstrate that t-hPSCs with depleted Oct4 retain pluripotency and TIC capacity and indicate that the enhanced tumorigenesis of t-hPSCs [30] is not dependent on Oct4. These functional results question previous notions that retention of Oct4 alone can be used as a functional indicator of hSC transformation [19], [37], [38].

### t-hPSCs Are Dependent on Nanog for Survival and Self-Renewal

**Figure 3:**
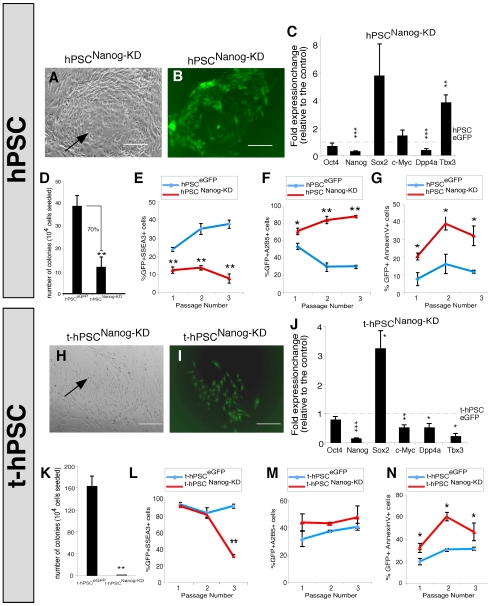
t-hPSCs exhibit a heightened self-renewal and survival response following Nanog dysregulation. (A–B) Representative images of normal hPSC bulk culture one week after transduction with a lentivector carrying an shRNA sequence targeting Nanog. Scale bar = 100 µm, n = 5. A: Phase contrast. B: GFP. Note the less compact colony morphology and the differentiated interior. Arrow denotes differentiated cells in the hPSC colony centre following Nanog knockdown. (C) qPCR demonstrating fold changes in Oct4, Nanog, Sox2, c-Myc, Dpp4a, and Tbx3 transcripts in GFP^+^SSEA3^+^ cells isolated from Nanog knockdown hPSCs relative to the control, respectively. Bar graphs represent mean values ± SEM, n = 3. **, p<0.01, ***, p<0.001. (D) Clonogenic self-renewal of SSEA3^+^ cells isolated from control and Nanog knockdown hPSCs. 1×10^4^ GFP^+^SSEA3^+^ cells were isolated from hPSCs 2 days after transduction with eGFP control and Nanog knockdown lentiviral vectors and seeded on ihdFs. GFP^+^ colonies were scored 9 days after seeding. Bar graphs represent mean values ± SEM, n = 3. **, p<0.01. (E–G) Frequency of GFP^+^SSEA3^+^ (E) GFP+A2B5+ (F) and GFP+AnnexinV+ (G) cells for three passages of culture derived from sorted GFP^+^SSEA3^+^ fractions of control and Nanog knockdown hPSCs. Lines represent mean values ± SEM, n = 3. * p<0.05, ** p<0.01. (H–I) Representative images of t-hPSC bulk culture one week after transduction with a lentivector carrying an shRNA sequence targeting Nanog. Scale bar = 100 µm, n = 5. H: Phase contrast. I: GFP. Arrow denotes t-hPSCs undergoing apoptosis following Nanog downregulation. (J) qPCR demonstrating fold changes in Oct4, Nanog, Sox2, c-Myc, Dpp4a, and Tbx3 transcript in GFP^+^ cells isolated from Nanog knockdown t-hPSCs. Bar graphs represent mean values ± SEM, n = 3, *, p<0.05, **, p<0.01, ***, p<0.001. (K) Clonogenic self-renewal of GFP^+^ cells isolated from control and Nanog knockdown t-hPSCs. 1×10^4^ were sorted from t-hPSCs 4 days after transduction with eGFP control and Nanog knockdown lentiviral vectors and seeded on ihdFs. GFP^+^ colonies were scored 6 days after seeding. Bar graphs represent mean values ± SEM, n = 3. **, p<0.01. (L–N) Frequency of GFP^+^SSEA3^+^ (L) GFP+A2B5+ (M) and GFP+AnnexinV+ (N) cells for three passages of control and Nanog knockdown t-hPSCs. Lines represent mean values ± SEM, n = 3. * p<0.05.** p<0.01.

In addition to Oct4, Nanog has also been established as a core pluripotency factor [13], [14]. However, the role of Nanog in SC transformation is unknown. To determine the functional relevance of Nanog expression, we stably and effectively knocked down Nanog using shRNA in both hPSCs and t-hPSCs (Figure S1U). Consistent with previous reports [31], Nanog depletion resulted in the differentiation of colonies in normal hPSC cultures demonstrating that this TF is required for normal pluripotent SC cell maintenance (Figure 3A–B). Since normal hPSCs underwent differentiation, we then isolated transduced GFP+ hPSCs expressing the primitive marker SSEA3 to investigate the specific effect of Nanog depletion on the self-renewing clonogenic fraction.

*Nanog* and *Oct4* co-occupy target genes and form specialized autoregulatory and feedforward loops to establish molecular control of ESC pluripotency [12], [39]. To evaluate the molecular mechanisms responsible for the functional changes in clonogenic hPSCs following Nanog knockdown, we looked at transcript levels of genes associated with hPSC pluripotency. While shRNA-based Nanog depletion decreased Dpp4a expression, both Oct4 and c-Myc levels remained unchanged in normal hPSCs (Figure 3C). In contrast, Tbx3 transcript was significantly upregulated along with an increase in Sox2 levels (Figure 3C). The similar gene expression patterns following both Oct4 and Nanog downregulation in hPSCs confirm previous studies demonstrating that these TFs share several targets [12].

To characterize the relevance of Nanog dysregulation to hPSC function, we examined the effect of Nanog reduction on hPSC clonogenic self-renewal, differentiation and survival. Similar to Oct4, Nanog downregulation also significantly decreased the number of colonies generated from the SSEA3+ subset as compared to controls (Figure 3D). This indicates a critical role for Nanog in the clonogenic self-renewal of normal hPSCs. This reduction in self-renewal potential was consistent with the loss in SSEA3 over passage and was also accompanied by an increase in the expression of the neural marker A2B5 [30] demonstrating a role for Nanog in preventing differentiation (Figure 3E–F). Additionally, Nanog knockdown induced apoptosis represented by an increased frequency of Annexin V^+^ cells (Figure 3G). Together, these results show that Nanog is critical in maintaining the undifferentiated state of normal hPSCs while repressing both neural differentiation and apoptosis.

Transformed-hPSCs expressed only slightly higher levels of Nanog than normal hPSCs. (Figure S1 V). Given the lack of effect of Oct4 depletion on transformed cells, we sought to investigate whether Nanog regulates t-hPSC function. Nanog knockdown induced differentiation and compromised viability in t-hPSCs bulk compared to eGFP controls (Figure 3H–I). To evaluate the molecular mechanisms associated with these biological changes, we isolated GFP^+^ cells from Nanog-depleted t-hPSCs and examined transcript levels of Oct4, Nanog, Sox2, c-Myc, Dpp4a and Tbx3 compared with cells transduced with the control eGFP vector. Relative to normal hPSCs, Nanog knockdown in t-hPSCs resulted in similar patterns of Nanog, Oct4, Sox2, and Dpp4a transcript regulation (Figure 3J). However, significant decreases in both c-Myc and Tbx3 (Figure 3J) demonstrate that Nanog differentially regulates transcriptional networks in t-hPSCs compared with normal cells.

To understand the potential biological effects of Nanog depletion on the self-renewing fraction, transduced t-hPSCs were selected and cultured to evaluate effects on self-renewal, differentiation and apoptosis. Surprisingly, Nanog downregulation completely abolished colony formation capacity (Figure 3K) revealing an obligatory role for Nanog in the clonogenic self-renewal unique to t-hPSCs vs. normal hPSCs. Since colonies could not be recovered following Nanog depletion in t-hPSCs, we measured the effect of Nanog downregulation on t-hPSC differentiation using GFP+ cells from transduced bulk culture. SSEA3 levels were significantly reduced after 3 passages, however, there was no change in frequency of cells expressing A2B5 (Figure 3L–M). This demonstrated that unlike normal hPSCs, Nanog does not regulate t-hPSC neural differentiation. t-hPSCs also underwent a significant apoptotic induction shown by an increased frequency of Annexin V^+^ cells (Figure 3N). Together, these results demonstrate a potent and distinct hypersensitivity of t-hPSCs to Nanog.

### Nanog Regulation of Apoptosis in t-hPSCs Is Oct4 Dependent

**Figure 4:**
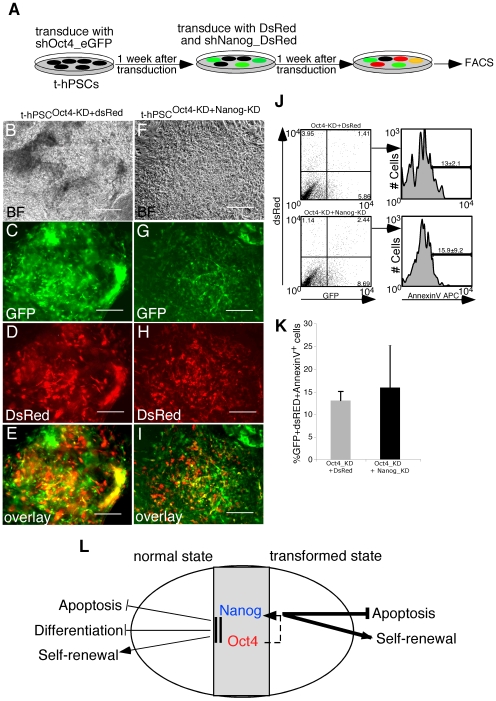
Survival of t-hPSCs after dual and sequential knockdown of Oct4 and Nanog. (A) Schematic showing the protocol for dual and sequential knockdown of Oct4 and Nanog in t-hPSCs. (B–I) Representative images of control (B–E) and dual Oct4 and Nanog knockdown (F–I) t-hPSCs 2 weeks after transduction with the shOct4_GFP vector followed by control DsRed and shNanog_DsRed vectors, respectively. Scale bar = 100 µm, n = 3. Images are representative of individual colonies seen throughout the entire culture. Note the significant overlay between Oct4 KD-dsRED (E) and Oct4 KD-Nanog KD (I) demonstrating that Oct4 and Nanog are downregulated in the same cells. (J) Representative FACS results showing the frequencies of Annexin V^+^ cells within GFP^+^DsRed^+^ fractions of control and dual Oct4 and Nanog knockdown t-hPSCs. n = 3. (K) Frequency of Annexin V^+^ cells within GFP^+^DsRed^+^ fractions of control and dual Oct4 and Nanog knockdown t-hPSCs. (13±2.1% AnnexinV+ cells in Oct4 KD/dsRED t-hPSC controls vs 15.9±9.2% Annexin V+ cells in Oct4 KD/Nanog KD-dsRED t-hPSCs. Bar graphs represent mean values ± SEM, n = 3, p>0.05. (L) While both Oct4 and Nanog are required to sustain the normal human stem cell pluripotent state, Oct4 is dispensable for transformed human stem cell self-renewal and survival. However, transformed human stem cells are completely dependent on Nanog for both self-renewal and survival revealing a fundamental paradigm shift in the role of core TFs following transformation. This heightened effect of Nanog on transformed cell survival is dependent on Oct4.

Although Nanog downregulation induced cell death in t-hPSCs (Figure 3N), Nanog transcript downregulation following Oct4 knockdown did not induce a similar apoptotic response in t-hPSCs (Figure 1N). This suggests that Nanog-regulated survival is Oct4 dependent. To examine the specific functional relationship of Oct4 and Nanog in transformed hSCs, we performed both dual and sequential knockdown of Oct4 and Nanog in t-hPSCs using the approach depicted (Figure 4A). Consistent with our previous results, initial Oct4 knockdown had no effect on t-hPSC differentiation (Figure 4B–C,F–G and data not shown). Surprisingly, dual Oct4 and Nanog knockdown t-hPSCs survived compared with control t-hPSCs (Figure 4D–E, H–I). The potent apoptotic effect seen in Nanog-knockdown only transformed cells (Figure 3N) was completely abolished when sequential knockdown of Oct4 and Nanog was performed (Figure 4J–K). This demonstrates that Nanog–regulated apoptosis is dependent on Oct4. Our results reveal a hierarchical role for Nanog and Oct4 in t-hPSC regulation.

## Discussion

The core transcription factors that regulate self-renewal, survival and developmental potential are well established in hPSCs [12]. This provides a unique opportunity to determine the role of these governing factors in the normal vs. transformed SC state in a manner that cannot be fully evaluated in other CSC systems limited by patient sample heterogeneity and availability. Our study defines a mechanistic distinction of Oct4 and Nanog in transformed vs. normal hSCs. While embryonic gene expression patterns have recently been associated with malignancy [16], [17], [18], we provide evidence that Oct4 alone may have unique functions in normal SCs, whereas transformed SCs are strongly dependent on Nanog. Based on our results, we propose a model to describe the functional role and relationship of Oct4 and Nanog in transformed vs. normal SC state (Figure 4L).

Differences that separate normal vs. cancer SC molecular circuitry are not well characterized and therefore hinder development of novel therapeutics that specifically target CSCs. A reduction in Oct4 and Nanog levels induces spontaneous lineage development and loss of pluripotent self-renewal capacity in hPSCs [11], [31], [32]. Unlike normal SCs, Oct4 is dispensable for self-renewal, survival and differentiation of transformed cells (Figure 4L). In contrast, Nanog represents a t-hPSC “achilles heel”, as the strong survival effect combined with the abolishment of clonogenic self-renewal reveals a fundamental dependence on a single TF for cellular maintenance in the transformed state. The role for Nanog in t-hPSC survival was dependent on Oct4, as evidenced by abolishment of the apoptotic effect following dual knockdown. The inherent vulnerability of t-hPSCs to Nanog suggests that functional characterization of TFs governing the pluripotent state may reveal unique dependencies of SCs upon entry into neoplastic, transformed states of self-renewal.

While Oct4 plays a regulatory role in t-hPSC survival, our work also indicates that Oct4 expression is not a relevant criterion to pathologically define transformation of hPSCs *in vitr*o or *in vivo*. This is supported by evidence demonstrating that Oct4 is not detected in a panel of nearly 200 solid tumors [19] and is dispensable for the maintenance of adult mammalian somatic SCs [40]. Prior to these functional TF studies, the overexpression of Oct4 in cultured t-hPSCs combined with the presence of Oct4-positive pluripotent cells in teratomas [30] would have been misconstrued as indicators of malignant progression of hPSCs. As such, differential expression of core TF genes does not necessarily link SCs with cancer, thus underscoring the need for functional validation of all potential biomarkers. Nevertheless, the association of embryonic gene expression patterns with malignancy has recently gained considerable momentum [16], [17], [18]. Combined with our results, this implies that tumor cells acquire heightened self-renewal capacity by hijacking TFs typically associated with hPSCs, or that CSC populations may utilize pluripotent TFs for tumor maintenance. Involvement of Oct4 and Nanog in either process would allow one to capitalize on these functional dependencies and target TFs therapeutically.

TFs are critical regulators of normal SC and cancer cell self-renewal, survival and differentiation. While similar gene expression signatures are suggestive of a tumor stem-cell phenotype, a more complete understanding of the mechanisms behind these potential biological indicators or markers is essential. Without this, treatment strategies targeting specific TFs could have vastly different and unexpected effects on a patient's cancer/CSC vs. normal SC population. Our work reveals a functional divergence of transcriptional machinery from the normal SC self-renewing state versus transformation. In light of recent studies demonstrating a role for Oct4 and Nanog in tumor progression [15], [20], this mechanistic distinction may not be exclusive to hPSCs, but more broadly applicable to multiple CSC types. The divergent roles of Oct and Nanog revealed in this study establish a paradigm to develop novel therapeutics towards selective destruction of aggressive tumors harboring CSCs with similar molecular signatures.

## Materials and Methods

### Ethics Statement

All animal experiments were approved by the local authority, the Animal Care Council and Veterinary Services of McMaster University.

### Culture of hPSCs and t-hPSCs, and Formation of hEBs

H9 and H1 hPSC lines as well as the H9-derived t-hPSC line were cultured as previously described [30]. Briefly, all cell lines were cultured on Matrigel (BD Biosciences) coated plates and maintained in mouse embryonic fibroblast conditioned medium (MEF-CM) supplemented with 8 ng/ml of human recombinant basic fibroblast growth factor (bFGF, Invitrogen) [41]. Formation of hEBs from t-hPSCs and hematopoietic differentiation of hEBs were performed as previously reported [30], [41].

### Lentiviral shRNA Vector Subcloning

Construction of the lentiviral vector Lentilox3.7 (LL3.7) carrying the eGFP reporter was performed as described [42]. An oligonucleotide targeting the human *Nanog* gene and two oligonucleotides targeting the human *Oct4/POU5F1* gene were designed and generated [31]. The third oligonucleotide encoding stem-loop structures targeting the human *Oct4/POU5F1* gene with the targeting sequence AATTGCTCGAGTTCTTTCT was designed using the Darmacon company siRNA design tool (http://www.dharmacon.com/DesignCenter/DesignCenterPage.aspx). These oligonucleotides were subcloned into the LL3.7 vector under the control of the U6 promoter. DsRed was subcloned into the LL3.7 control vector to replace eGFP. Briefly, DsRed was amplified with primers (forward-AATTCGCTAGCCGCCACCATGGCCTCCTCC: reverse-TCGAGGAATTCCTACAGGAACAGGTGGTGGCGGC) including Nhe1 and EcoR1 as restriction sites respectively and was inserted into LL3.7 vector by replacing eGFP sequences. The oligonucleotide targeting the human *Nanog* gene was also subcloned into the engineered lentiviral vector carrying DsRed as the reporter. All engineered lentiviral vectors were verified by sequencing.

### Lentiviral Virus Production and hPSCs Transduction

Lentiviruses were produced in 293FT cells (ATCC) as described [42], [43]. Briefly, lentiviral vectors were co-transfected with the third generation packaging plasmids encoding gag/pol, REV and vesticular stomatitis virus G protein at a ratio 2∶1 by lipofectamine 2000 (Invitrogen) into 293FT cells (DNA/lipofectamine = 1 µg/3µl). Viral supernatants were collected 72 hours after transfection and concentrated by ultracentrifugation to produce stock with titers of 4.8×10^7^ to 8.1×10^7^ infectious units per milliliter. Virus titers were determined on Hela cells. To transduce normal hPSCs and t-hPSCs, 1.8×10^7^ hPSCs and 1.5×10^6^ t-hPSCs on day 1 after passage were transduced with viruses in MEF-CM supplemented with 8 ng/ml bFGF and 8 ng/ml polybrene (Chemicon international) for 24 hours. Multiplicities of infection (MOI) of 0.1 and 1 were used to transduce the cells with LL3.7_eGFP, LL3.7_DsRed, LLshOct4-1_eGFP, LLshOct4-2_eGFP, LLshOct4-3_GFP, LLshNanog_eGFP, LLshNanog-DsRed lentiviral vectors.

### Isolation of hPSCs for Clonogencity Analysis

Transduced hPSCs and t-hPSCs were isolated using a FACSAria (BD Biosciences) and re-plated for the clonal assay previously described [33]. Briefly, hPSCs were dissociated on day 2 after lentiviral transduction and stained with SSEA3 (Develop Studies Hybridoma Bank, mAB clone MC-631) and secondary AlexaFluor-647-goat-anti-mouse-IgG (Molecular Probes). 1×10^4^ 7AAD^−^GFP^+^SSEA3^+^ cells were sorted with 94–98% purity and seeded on 12-well tissue culture plates coated with irradiated hPSC-derived fibroblast-like cells (ihdFs). 9 days after seeding, the number of GFP^+^ colonies was counted under fluorescent microscope (Olympus). t-hPSCs were dissociated 4 days after transduction and 7AAD^−^GFP^+^ t-hPSCs were sorted and plated at cell doses of 1×10^4^, 1×10^3^, 1×10^2^ and 10 on 12-well and 96-well tissue culture plates coated with ihdFs. On day 6, total number of GFP^+^ colonies derived from t-hPSCs was counted under fluorescent microscope. Sorted hPSCs and t-hPSCs were expanded for other assays.

### Flow Cytometry Analysis of Oct4, SSEA3, A2B5 and Annexin V

hPSCs and t-hPSCs were treated with Collagenase IV for 7 to 10 minutes followed by cell dissociation buffer (Gibco) for 10 minutes at 37°C and filtration through a 40 µm cell strainer. For Oct4 staining, cells were fixed and stained with mouse anti-oct3-MAb (Beckton Dickinson), followed by secondary staining with either Alexa fluor 647 goat anti mouse IgG (Invitrogen) or goat F (ab') 2 fragment anti-mouse IgG (H+L) PE. For SSEA3 staining, we used SSEA3 (Develop Studies Hybridoma Bank, mAb clone MC-631) and goat F(ab')2 fragment anti-mouse IgG (H+L) PE or FITC (Invitrogen) or Alexa fluor 647 goat anti mouse IgG (Invitrogen). A2B5 was detected with antibodies A2B5 (R&D systems) and Alexa fluor 647 goat anti mouse IgM (Invitrogen). Live cells were identified by 7-aminoactinomycin D (7-AAD) exclusion and analyzed for surface-marker expression using FACS Calibur (BD Biosciences). The data were analyzed by FlowJo software (Tree Star). The apoptotic status of the cells was assessed using the AnnexinV apoptosis detection kit (BD Biosciences) according to the manufacturer's guidelines.

### Teratoma Formation

1.5×10^4^, 3.3×10^4^, 6.6×10^4^ sorted Oct4 knockdown t-hPSCs were injected into the testis capsules of three male NOD/SCID mice in triplicate. Cell doses of 7.5×10^3^, 2.4×10^4^, and 6.6×10^4^ t-hPSCs were also injected into 6 mice as control [30]. After 6 weeks, teratomas were extracted, measured and fixed with 10% buffered formalin followed by embedding in paraffin. Five micron-sectioned samples were stained with Hematoxalin and Eosin (H&E) and imaged under 200× magnification.

### Quantitative Polymerase Chain Reaction (qPCR) Analysis

Total RNA from hPSCs and t-hPSCs was extracted by RNeasy Mini Kit (Qiagen) according to the manufacturer's instructions. cDNA synthesis was performed with 5 µg total RNA using by first-strand cDNA synthesis kit (Amersham Biosciences). Expression of Oct4, Nanog, Sox2, c-Myc, Dpp4a, and Tbx3 were quantified by quantitative PCR (Mx4000, Stratagene) using SYBR green (Invitrogen) DNA-binding dye. Quantitative PCR reaction conditions were as follows: Primary denaturation at 95°C for 1 min and 40 cycles of PCR consisting of 95°C for 10 s, 60°C for 1 min, and 72°C for 30 s, followed by analyzing the amplified products using the dissociation curves. The signal intensities were normalized against GAPDH and the 2^−ΔΔCt^ equation was used to calculate the relative gene expressions [44]. The primers used are listed in Table S2.

### Statistical Analysis

Results were presented as mean ± SEM. Statistical significance was determined using an unpaired Student *t* test and results were considered significant or highly significant when *p*≤0.05 or ≤0.01, respectively.

## Supporting Information

Figure S1 Lentivirus-based Oct4 and Nanog shRNA significantly downregulate Oct4 and Nanog expression, respectively, in both normal hPSCs and t-hPSCs. (A-B) Representative FACS histograms of Oct4+ cell frequency within gated GFP^+^SSEA3^+^ fractions from control (A) and Oct4 knockdown (B) hPSCs. (C-D) Frequency (C) and mean fluorescence intensity (D) of Oct4+ cells within gated GFP^+^SSEA3^+^ fractions from control and Oct4 knockdown hPSCs. Error bars represent SEM, n = 3. (E-F) Representative FACS histograms of Oct4+ cell frequency within gated GFP+ fraction from control (E) and Oct4 knockdown (F) t-hPSCs. (G-H) Frequency (G) and mean fluorescence intensity (H) of Oct4+ cells within the gated GFP+ fraction from control and Oct4 knockdown t-hPSCs. Error bars represent SEM, n = 5. (I-L) Representative images of bulk H1 and H9 hPSCs one week after transduction with the control lentilox vector LL3.7 (eGFP as transduction reporter, hPSCeGFP) (I-J) or the Oct4 knockdown lentiviral vector (K and L). Scale bar = 100 µm, n = 5. I and K: Phase contrast. J and L: GFP. (M-P) Representative images of bulk H9 t-hPSCs one week following transduction with control (M and N) and Oct4 knockdown lentiviral vectors (O and P). n = 5. M and O: Phase contrast. N and P: GFP. Scale bar = 100 µm. (Q-T) Representative images of control (Q and R) and Oct4 knockdown (S and T) t-hPSC cultures 4 months after sorting GFP+ cells. Scale bar = 100 µm. (U) qPCR of fold changes in Nanog transcripts following stable Nanog knockdown (black bars) in both hPSCs and t-hPSCs relative to transduction with the control eGFP lentivirus (white bars). Bar graphs represent mean values ± SEM, n = 3, ***, p<0.001. (V) qPCR results showing the fold change of t-hPSC Nanog transcript relative to normal hPSCs. Bar graphs represent mean values ± SEM, n = 3.
(2.32 MB PDF)

Figure S2 Schematic of GFP^+^SSEA3^+^ and GFP+ fractions isolation from control and Oct4 knockdown normal hPSCs and t-hPSCs respectively. Sorted cells were seeded at clonal density on irradiated hdFs and sorting purities for each fraction are shown.
(0.02 MB PDF)

Table S1 Limiting dilution assay for teratoma formation from control and Oct4 knockdown t-hPSCs.
(0.00 MB PDF)

Table S2 Primers used in quantitative real-time reverse transcription-polymerase chain reaction studies.
(0.25 MB PDF)
