# Supplementary figures and images for: Pluripotent Transcription Factors Possess Distinct Roles in Normal versus Transformed Human Stem Cells
Source: PLoS One. 2009 Nov 30;4(11):e8065. doi: 10.1371/journal.pone.0008065 (PMC2778551; doi:10.1371/journal.pone.0008065)

Ji et al., Supplemental Figure 1

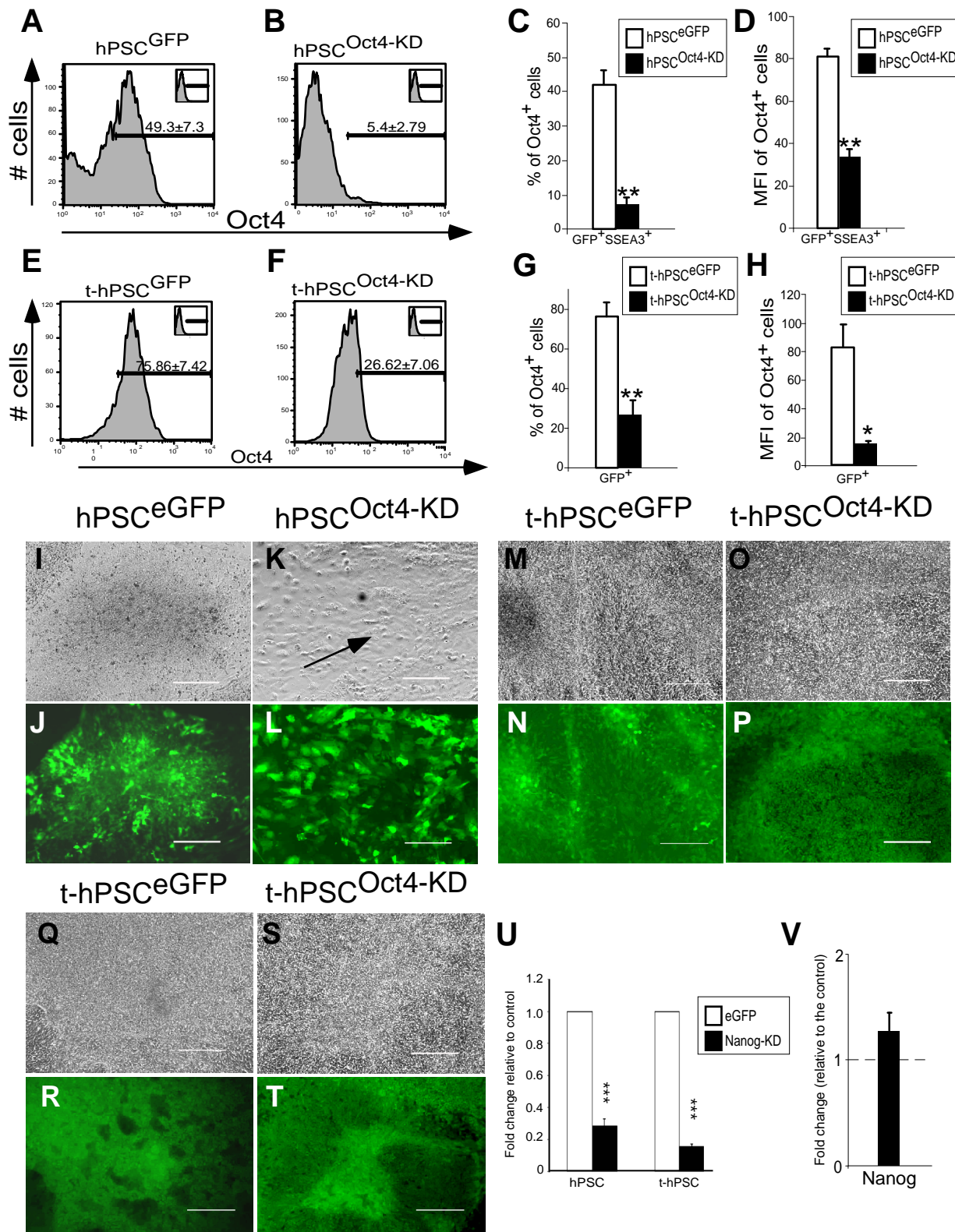

Supplement: Figure S1 — Lentivirus-based Oct4 and Nanog shRNA significantly downregulate Oct4 and Nanog expression, respectively, in both normal hPSCs and t-hPSCs. (A-B) Representative FACS histograms of Oct4+ cell frequency within gated GFP+SSEA3+ fractions from control (A) and Oct4 knockdown (B) hPSCs. (C-D) Frequency (C) and mean fluorescence intensity (D) of Oct4+ cells within gated GFP+SSEA3+ fractions from control and Oct4 knockdown hPSCs. Error bars represent SEM, n = 3. (E-F) Representative FACS histograms of Oct4+ cell frequency within gated GFP+ fraction from control (E) and Oct4 knockdown (F) t-hPSCs. (G-H) Frequency (G) and mean fluorescence intensity (H) of Oct4+ cells within the gated GFP+ fraction from control and Oct4 knockdown t-hPSCs. Error bars represent SEM, n = 5. (I-L) Representative images of bulk H1 and H9 hPSCs one week after transduction with the control lentilox vector LL3.7 (eGFP as transduction reporter, hPSCeGFP) (I-J) or the Oct4 knockdown lentiviral vector (K and L). Scale bar = 100 µm, n = 5. I and K: Phase contrast. J and L: GFP. (M-P) Representative images of bulk H9 t-hPSCs one week following transduction with control (M and N) and Oct4 knockdown lentiviral vectors (O and P). n = 5. M and O: Phase contrast. N and P: GFP. Scale bar = 100 µm. (Q-T) Representative images of control (Q and R) and Oct4 knockdown (S and T) t-hPSC cultures 4 months after sorting GFP+ cells. Scale bar = 100 µm. (U) qPCR of fold changes in Nanog transcripts following stable Nanog knockdown (black bars) in both hPSCs and t-hPSCs relative to transduction with the control eGFP lentivirus (white bars). Bar graphs represent mean values ± SEM, n = 3, ***, p<0.001. (V) qPCR results showing the fold change of t-hPSC Nanog transcript relative to normal hPSCs. Bar graphs represent mean values ± SEM, n = 3. (2.32 MB PDF) [file pone.0008065.s001.pdf]

Ji et al., Supplementary Figure 2

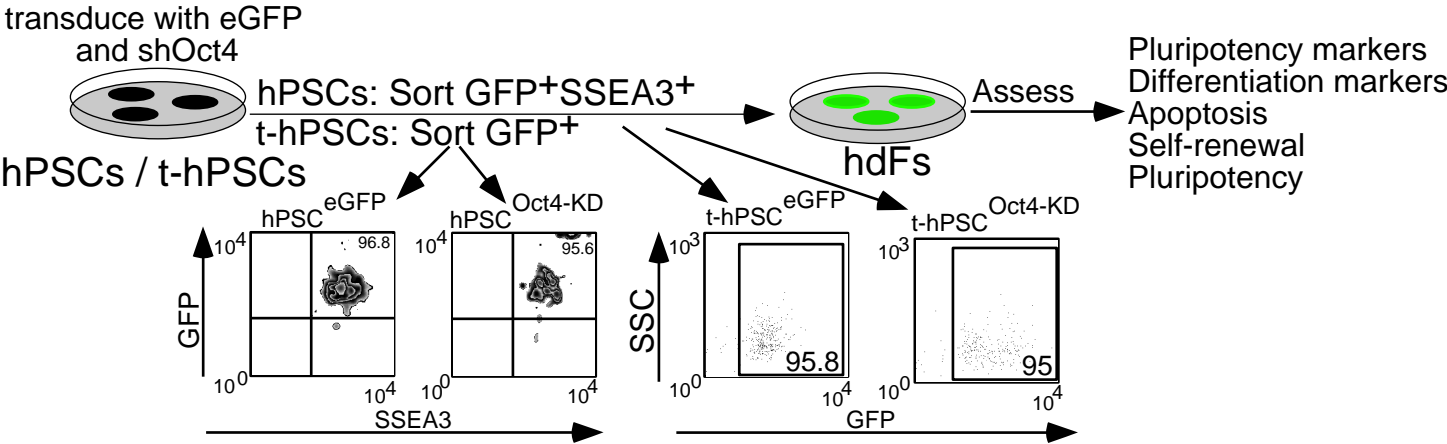

Supplement: Figure S2 — Schematic of GFP+SSEA3+ and GFP+ fractions isolation from control and Oct4 knockdown normal hPSCs and t-hPSCs respectively. Sorted cells were seeded at clonal density on irradiated hdFs and sorting purities for each fraction are shown. (0.02 MB PDF) [file pone.0008065.s002.pdf]
