# Supplementary material for: Pluripotent Transcription Factors Possess Distinct Roles in Normal versus Transformed Human Stem Cells
Source: PLoS One. 2009 Nov 30;4(11):e8065. doi: 10.1371/journal.pone.0008065 (PMC2778551; doi:10.1371/journal.pone.0008065)
Supplement: Table S1 — Limiting dilution assay for teratoma formation from control and Oct4 knockdown t-hPSCs. (0.00 MB PDF) [file pone.0008065.s003.pdf]

Ji *et al.*, Supplemental Table 1

Limiting dilution assay for t-hPSC$^{Oct4-KD}$ teratoma formation

Mouse ID	Cell info	Number of cells injected ($x10^3$)	Volume of mass (cm^3)	Histology (HE)	Metastases	Lymphoma
1#	t-hPSC$^{Oct4-KD}$	66	0.13	2 germ layers	No	No
2#	t-hPSC$^{Oct4-KD}$	66	13.00	3 germ layers	No	No
3#	t-hPSC$^{Oct4-KD}$	66	3.00	3 germ layers	No	No
4#	t-hPSC$^{Oct4-KD}$	33	2.40	3 germ layers	No	No
5#	t-hPSC$^{Oct4-KD}$	33	3.74	3 germ layers	No	No
6#	t-hPSC$^{Oct4-KD}$	33	8.64	3 germ layers	No	No
7#	t-hPSC$^{Oct4-KD}$	15	1.60	3 germ layers	No	No
8#	t-hPSC$^{Oct4-KD}$	15	1.98	3 germ layers	No	No
9#	t-hPSC$^{Oct4-KD}$	15	9.60	2 germ layers	No	No

Note: Control testes are normal and around 0.14g
