# Supplementary material for: Pluripotent Transcription Factors Possess Distinct Roles in Normal versus Transformed Human Stem Cells
Source: PLoS One. 2009 Nov 30;4(11):e8065. doi: 10.1371/journal.pone.0008065 (PMC2778551; doi:10.1371/journal.pone.0008065)
Supplement: Table S2 — Primers used in quantitative real-time reverse transcription-polymerase chain reaction studies. (0.25 MB PDF) [file pone.0008065.s004.pdf]

Ji *et al.*, Supplemental Table 2

Primers used in quantitative real-time reverse transcription-polymerase chain reaction studies

Target gene	NCBI accession number	Forward & Reverse primers
oct4/pou5f1	NM_002701	CTGAAGCAGAAGAGGATCAC<br>GACCACATCCTTCTCGAGCC
nanog	NM_024865	CGAAGAATAGCAATGGTGTGACG<br>TTCCAAAGCAGCCTCCAAGTC
c-myc	NM_002467.3	AGCGACTCTGAGGAGGAACAACAA<br>AGAAGGTGATCCAGACTCTGACCT
sox2	NM_003106	CGAGATAAACATGGCAATCAAATG<br>AACGTTTGCCTTAAACAAGACCAC
dppa4	NM_018189	ACCTCAGAAGAAGATACCAATCC<br>AAGGCACACAGGCGCTTA
tbx3	NM_005996.3	TCCATGAGGGTGTTTGATGA<br>CGCTGGGACATAAATCTTTGA
GAPDH	NM_014364	TGCACCACCAACTGCTTAGC<br>GGCATGGACTGTGGTCATGAG
